# A pilot study of spinal reflex modulation during immersive virtual reality exposure in humans

**DOI:** 10.3389/fnhum.2026.1872848

**Published:** 2026-08-05

**Authors:** Maxim Baltin, Anna Shulman, Margarita Nikulina, Elizaveta Vinogradova, Dmitry Onishchenko, Diana Sabirova, Tatyana Baltina

**Affiliations:** 1 Research Center for Genetics and Life Sciences, Sirius University of Science and Technology, Sirius Federal Territory, Russia; 2 Research Laboratory of Mechanobiology, Institute of Fundamental Medicine and Biology, Kazan Federal University, Kazan, Russia; 3 Laboratory of Microbiome, Center for Life Sciences, National Laboratory Astana, Nazarbayev University, Astana, Kazakhstan

**Keywords:** H-reflex, Jendrassik maneuver, M-response, neuromodulation, segmental excitability, spinal reflexes, virtual reality

## Abstract

**Background:**

The integration of virtual reality (VR) technologies into clinical rehabilitation and diagnostics is rapidly expanding, yet the neurophysiological mechanisms of their influence on spinal motor circuits remain poorly understood. This pilot study assessed the modulation of spinal reflex excitability during exposure to specific immersive audiovisual stimuli delivered via a virtual reality headset, compared to classical neuromodulation paradigm.

**Materials and methods:**

Twenty-one healthy volunteers completed a within-subject design. The H-reflex and M-response parameters of the *soleus* muscle were recorded at rest, during the Jendrassik maneuver, and in three VR scenarios (“roller coaster,” “horror,” and “relaxation”). Autonomic responses were assessed using RR interval duration and RMSSD. Statistical analysis was performed using nonparametric methods for repeated measures.

**Results:**

H-reflex thresholds remained stable across all conditions (*q* > 0.1). H-reflex amplitude was significantly modulated: the Jendrassik maneuver caused bilateral facilitation of the reflex (*q* = 0.10 and *q* = 0.09 for the right and left legs, respectively), while VR stimuli designed to elicit emotional responses induced suppression (a significant reduction was observed in the non-dominant leg, *q* = 0.06, with a similar non-significant trend in the dominant leg). M-response parameters remained unchanged, indicating the stability of peripheral conduction.

**Conclusion:**

These preliminary results suggest that the specific immersive audiovisual stimuli tested in this study can selectively modulate spinal reflex excitability via central descending pathways rather than peripheral mechanisms. The results of this preliminary research provide a foundation for the design of future investigations.

## Introduction

Virtual reality (VR) use in healthcare has recently increased markedly, with particularly strong uptake in physiotherapy, rehabilitation, and patient diagnosis and assessment (Bateni et al., 2024). VR systems are now applied across a range of clinical conditions. For example, in a single case report, immersive VR-based trainers were associated with enhanced motor recovery in a patient with central nervous system damage. VR has also been combined with neuromodulation techniques: virtual cycling paired with transcutaneous spinal stimulation improved motor and sensory functions in a patient with spinal cord injury (Chu et al., 2024). In clinical practice, VR is increasingly explored for vestibular rehabilitation, with systematic reviews supporting its utility in improving postural control and reducing vestibular symptoms (Xie et al., 2021).

Despite the growing adoption of VR-based delivery systems in clinical practice, the neurophysiological mechanisms by which specific immersive audiovisual content affects motor systems remain poorly understood. Earlier, to address this, we analyzed the effect of VR on human postural stability. The analysis showed that during VR immersion, postural regulation relied on an increased contribution of somatosensory information and the cerebellum, reflecting a more conscious mechanism of postural stabilization (Bikchentaeva et al., 2024).

A number of studies have shown that immersion in stressful VR scenes, such as a virtual fall or exposure to heights, can reduce the amplitude of the monosynaptic response (H-reflex) of the gastrocnemius muscle reflecting activation of descending inhibitory influences (Grosprêtre et al., 2023). However, these findings largely relate to postural control or anxiety states and do not extend to a broader range of emotional and motor stimuli.

Emerging evidence suggests that emotional and sensory stimuli in VR can modulate activity at both the spinal and suprasegmental levels, including descending supraspinal and brainstem regulatory pathways (Buetler et al., 2022; Hodgson et al., 2023). These effects are thought to arise from a complex interplay of autonomic responses, limbic emotion–motivation circuits, and corticospinal control. Nevertheless, an integrative model of how these mechanisms interact remains underdeveloped. Moreover, it is unclear how stable or lateralized these effects are, and whether responses differ between the dominant and non-dominant limbs (Xin et al., 2020).

Furthermore, it remains unclear which descending systems–sympathetic, corticospinal, or vestibulospinal–are engaged during virtual stimulus perception and how they might integrate. Specifically, we do not know how the emotional component of VR (e.g., horror vs. relaxing video content) modulates spinal motor centers. This modulation may involve presynaptic afferent inhibition and motoneuronal excitability (Knikou, 2008; Metz et al., 2023).

One of the most valid non-invasive approaches to assess the functional state of segmental motor circuits is to record the H-reflex and M-response. The H-reflex is considered the electrical analog of the monosynaptic tendon reflex. It reflects the excitability of the motoneuron pool and the efficiency of Ia afferent transmission. In contrast, the M-response reflects direct activation of motor axons and serves as a control for peripheral stimulation factors. Changes in H-reflex parameters are widely used to study sensorimotor integration, presynaptic inhibition, and neuroplastic processes. This applies both to healthy individuals and to those with various neurological conditions (Knikou, 2008; Eremeev et al., 2012; Kuck et al., 2018).

In clinical neurophysiology, classical interventions such as the Jendrassik maneuver and transcranial magnetic stimulation are traditionally used to assess spinal excitability modulation. These methods alter descending influences and presynaptic inhibition of Ia afferents (Knikou, 2008; Petrosyan et al., 2020; Bikchentaeva et al., 2022). VR exposure, however, differs fundamentally from these techniques. It simultaneously activates sensory, emotional, and autonomic systems. Consequently, VR may exert a more complex descending influence on spinal motor circuits. Through its immersive and multimodal design, VR can engage entire sensorimotor loops and emotional-motivational networks (Diemer et al., 2015; Ross, 2021; Reale et al., 2023).

Thus, despite the growing clinical use of VR, the nature and structure of the descending regulatory mechanisms engaged by immersive stimulation remain incompletely understood. A detailed analysis of H-reflex and M-response parameters—objective electrophysiological markers of segmental activity—may allow a quantitative assessment of how different VR exposures affect spinal motor centers. Accordingly, the aim of the present study was to evaluate the excitability of segmental motor structures under immersive VR and to compare these effects with those produced by classical methods of reflex modulation.

## Materials and methods

### Participants

We recruited 21 healthy volunteers (12 men, 9 women) aged 20 to 30 years (mean age 24.1 ± 2.9 years). Clinical examination confirmed no musculoskeletal, neurological, or psychiatric disorders. All participants were right-leg dominant. Limb dominance was determined via a standard interview. Each participant gave written informed consent before the experiment. The study protocol, including the consent procedure, was approved by the Ethics Committee of Sirius University of Science and Technology and by the local ethics committee of Kazan Federal University. The study followed the principles of the Helsinki Declaration. A self-assessment questionnaire was used to assess participants’ well-being after the virtual reality sessions. According to the questionnaire, all participants reported no negative feelings and a sense of well-being, demonstrating the tolerability of the VR approach used in the study.

We used a within-subject repeated-measures design. Each participant served as their own control. This approach reduces inter-individual variability in H-reflex parameters and increases statistical power compared with between-group designs.

We did not perform an *a priori* power analysis. Instead, we based the planned sample size on previous neurophysiological studies that examined spinal reflex excitability modulation under virtual simulation and classical facilitation paradigms (Grosprêtre et al., 2023). Those studies reported measurable within-subject effects with smaller or comparable sample sizes.

### Recording of H-reflex and M-response

We recorded evoked potentials using non-invasive, disposable cup electrodes placed on the skin. A conductive gel served as the electrolyte medium. We fixed the electrodes with medical adhesive tape and used a strap electrode as the ground. For recording, we used a belly-tendon electrode montage.

We stimulated the nerve with a bipolar fork electrode that had a fixed inter-electrode distance of 25 mm on a block. Skin preparation consisted of cleaning with alcohol and mild abrasion; we reduced impedance below 5 kΩ. To ensure a stable signal, we applied conductive gel.

Participants sat on a couch with their legs hanging freely, without touching the floor. We chose this posture deliberately: foot support against a surface creates additional support-related afferentation, which can modulate spinal neuronal circuits and reduce (or even suppress) the H-reflex. Participants were given clear instructions to maintain muscle relaxation throughout the procedure.

We applied electrical stimulation over the tibial nerve (*n. tibialis*) in the popliteal fossa to elicit H- and M-responses. We placed the bipolar stimulating electrode in the middle of the popliteal fossa with the cathode positioned proximally. We fixed the bipolar surface-recording electrodes over the belly of the soleus muscle (*m. soleus*).

Stimulation consisted of rectangular monopolar pulses of 1 ms duration delivered every 10 s. We increased the current from 1 mA to supramaximal levels for the M-response in 1-mA steps. To construct the H-reflex recruitment curve, we used increasing stimulus intensities; for the M-response, we used supramaximal stimuli. For the main experimental conditions, we selected the H-reflex amplitude corresponding to 20–30% of the individual Mmax. By anchoring the H-reflex measurement to this proportion of the participant’s own Mmax, we standardized the recording conditions across experimental sessions and minimized variability arising from shifts along the recruitment curve and fluctuations in motoneuron pool excitability.

**Figure 1:**
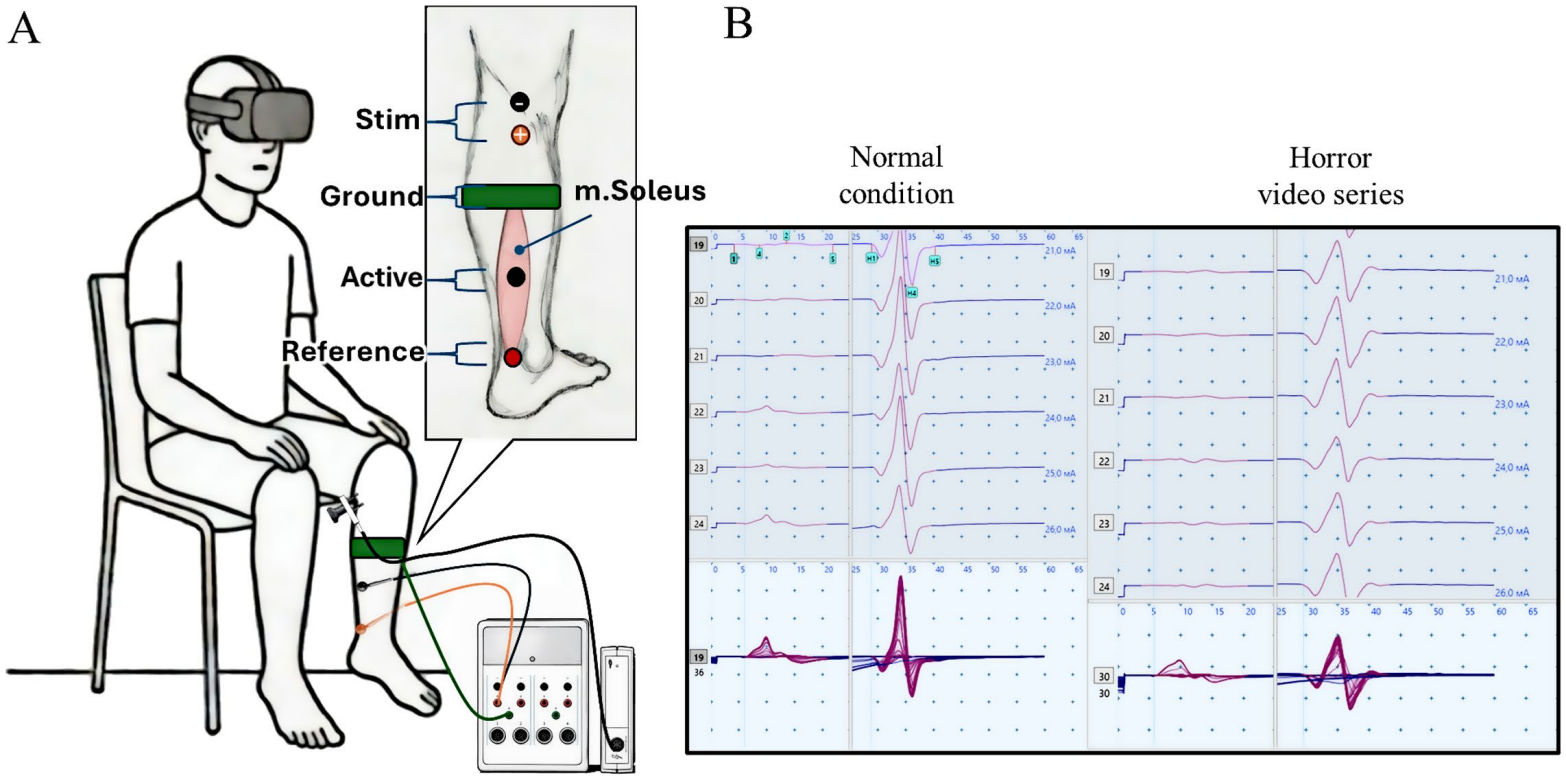
Schematic of the experiment. **(A)** Electrode placement. **(B)** Representative recordings of evoked potentials.

We recorded and processed the evoked biopotentials using a Neuro-MVP-4 electromyograph (Neurosoft, Russia). The analysis included the following parameters: H-reflex threshold—the minimum stimulus intensity required to activate the most excitable Ia afferents and their corresponding motoneurons, reflecting the level of segmental spinal excitability; M-response threshold—characterizing the excitability of distal motor axons (*α*-motoneurons), largely determined by peripheral mechanisms; H-reflex amplitude—indicating the efficiency of Ia afferent transmission onto α-motoneurons and serving as an integral measure of segmental spinal excitability; and M-response area—an integral measure accounting for both amplitude and duration, reflecting the total number of recruited motor units and being more resistant to desynchronisation and phase cancelation than peak amplitude (Figure 1).

### Experimental conditions

Each participant took part in a series of trials. The order of the trials was randomized using a Latin square design to exclude any order effects. First, we recorded H-responses and M-responses in the soleus muscle at rest. These data served as baseline controls. Subsequently, we recorded responses under the following conditions.

#### Jendrassik maneuver (JC)

The participant interlocked their fingers in front of the chest and then tried to pull their hands apart with maximum force. After 4 s of sustained isometric contraction with maximal effort, we recorded the evoked responses. This approach standardized the level of voluntary descending drive and minimized variability related to the force ramp-up phase.

#### Virtual reality (VR) exposure

Immersive audiovisual stimuli were delivered using an HTC Vive headset. All participants viewed identical, pre-recorded first-person video sequences that were strictly passive (non-interactive) in nature. Each condition lasted exactly 60 s. To account for initial visual-vestibular adaptation, electrophysiological recordings commenced 10 s after stimulus onset. Subsequent stimuli were delivered at 10-s intervals throughout the remaining 50-s window, yielding five averaged trials per condition. This standardized protocol ensured that all participants were exposed to the same content under identical technical and temporal conditions, allowing physiological responses to be attributed to the specific sensory and emotional characteristics of each scenario rather than to the VR medium itself.

- “Roller coaster” scenario (RC): Through the VR headset, the participant watched a dynamic first-person video of a roller coaster ride, immersing them in the amusement park experience.
- “Horror” scenario (Hor): The participant viewed a short video containing frightening visual elements, abrupt sounds, and jump-scare effects. The content was designed to incorporate such stimuli with the intent to provoke an effective response.
- “Relaxation” scenario (RX): The video featured meditative landscapes, nature sounds, and smooth music. This content was designed to promote a state of relaxation and reduction of emotional tension.

We kept a minimum interval of 10 min between conditions to allow neurophysiological parameters to return to baseline. We ensured identical lighting, noise levels, and body posture for all participants throughout the procedure. In all VR experimental conditions, we recorded H-waves and M-waves during continuous VR exposure while the participants remained motionless.

A self-assessment questionnaire was used to assess the participants’ well-being after the virtual reality sessions; according to the questionnaire, all participants reported no negative feelings and a feeling of well-being, indicating the tolerability of the VR approach used.

### Recording of RR intervals

Heart rate was recorded in all participants to assess autonomic responses to VR stimuli. A Polar chest-strap heart rate monitor was used with a sampling frequency of 1,000 Hz. The sensor was placed on the chest over the heart using a standard elastic belt and conductive gel. This ensured stable contact and minimized artifacts.

Heart rate recordings were synchronized with electromyography (Neuro-MVP-4 system, Neurosoft, Russia) using time stamps at the start of each experimental condition (VR scenarios and control interventions).

RR intervals were extracted automatically from the raw recording using Polar Sensor Logger software. The detection of R-peaks was then visually checked, and artefactual segments were excluded. Subsequent analyses were performed directly on the sequences of RR intervals.

The RMSSD index (Root Mean Square of Successive Differences) was also calculated. RMSSD is a time-domain measure of heart rate variability (HRV). It represents the square root of the mean squared differences between consecutive normal RR intervals (Berntson et al., 2005). RMSSD mainly reflects high-frequency heart rate fluctuations in the respiratory range (0.15–0.4 Hz). It is therefore considered an index of parasympathetic (vagal) cardiac regulation (Berntson et al., 2005; Begum, 2016).

### Statistical analysis

Data were analyzed with Python 3.12 using the following libraries: NumPy 2.0.1, SciPy 1.15.1, Matplotlib 3.10.0, and Seaborn 0.13.2. Group comparisons were performed using Friedman’s test and the Wilcoxon test. A Friedman test result with *p* < 0.05 was considered to indicate a significant change across conditions. When the Friedman test was significant, we conducted four paired comparisons between the control condition and the JC, RC, Hor, and RX conditions using the Wilcoxon test. A change was considered significant at *p* < 0.05. Additionally, we corrected both the omnibus and *post-hoc* tests using the FDR–BH procedure and explicitly reported the adjusted values for the better interpretation. Comparisons with q < 0.1 were considered significant after correction; all other comparisons were deemed insignificant after correction. Bar graphs were used for data visualization. Each bar graph displays the median and the 90% confidence interval (CI) around the median.

## Results

### Changes in *m. soleus* H-reflex parameters

**Figure 2:**
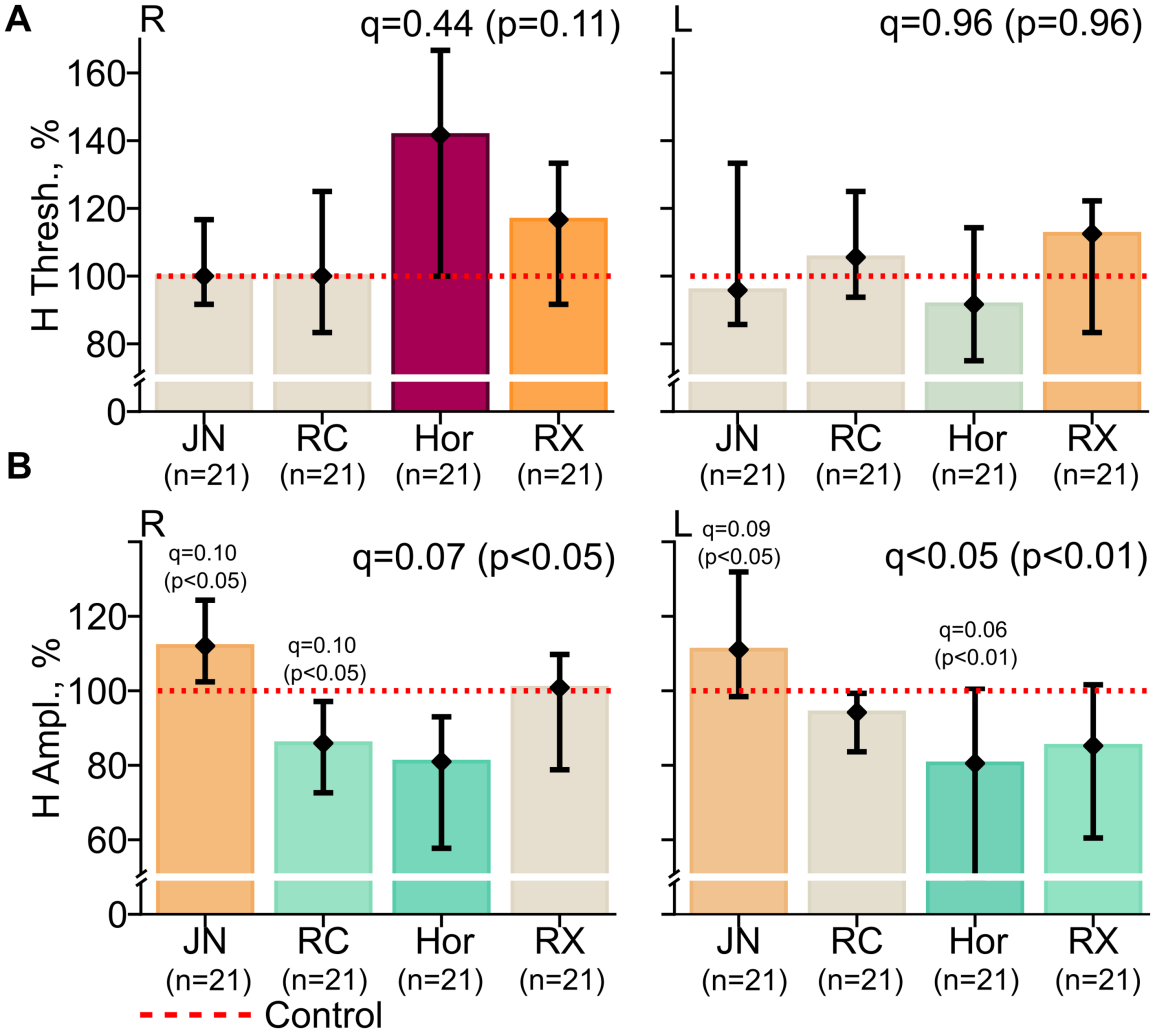
Threshold and area of the soleus H-reflex under different conditions for the right (R) and left (L) legs. **(A)** H-reflex threshold values of the *soleus* muscle. **(B)** Amplitude of the soleus H-reflex in percent (relative to control/rest condition). JN = Jendrassik maneuver (*n* = 21 participants); RC = virtual reality (VR) exposure—“Roller coaster” scenario (*n* = 21); Hor = VR “Horror” scenario (*n* = 21); RX = VR “Relaxation” scenario (*n* = 21); All comparisons included 21 participants.

No significant differences in the *soleus* H-reflex threshold between experimental conditions for either the right leg (*q* = 0.44, *p* = 0.11) or the left leg (*q* = 0.96, *p* = 0.96) were observed (Figure 2A). However, the right leg exhibited a trend toward the highest threshold value during the horror video condition. In the left leg, threshold values remained close to the control level across all conditions, with no clear trends.

Figure 2B shows a significant effect of the experimental conditions on H-response amplitude in the right leg (*q* = 0.07, *p* < 0.05). Compared to the control level, the Jendrassik maneuver significantly (*p* < 0.05) increased the H-response amplitude to 112.1% (90% CI: 102.2; 124.3). In contrast, viewing the roller coaster video decreased the H-response amplitude to 86.0% (90% CI: 72.8; 97.1) (*p* < 0.05). However, these observations did not remain significant after correction for multiple comparisons (*q* = 0.10 for both). Additionally, the horror video insignificantly (*p* > 0.05) decreased the H-response amplitude to 80.9% (90% CI: 57.7; 93.0).

In the left leg, the experimental conditions also had a significant effect (*q* < 0.05, *p* < 0.01). The Jendrassik maneuver increased (*p* < 0.05) the H-response amplitude to 111.1% (90% CI: 98.4; 130.4). The horror video decreased (*p* < 0.01) it to 80.6% (90% CI: 39.5; 99.4) compared to the control. These observations remained significant after correction for multiple comparisons (*q* = 0.09 and *q* = 0.06, respectively).

Thus, the Jendrassik maneuver facilitated the H-reflex in both legs. Conversely, the horror video significantly suppressed the H-response in the left leg, while a similar reduction in the right leg did not reach significance.

### Changes in soleus M-response parameters

**Figure 3:**
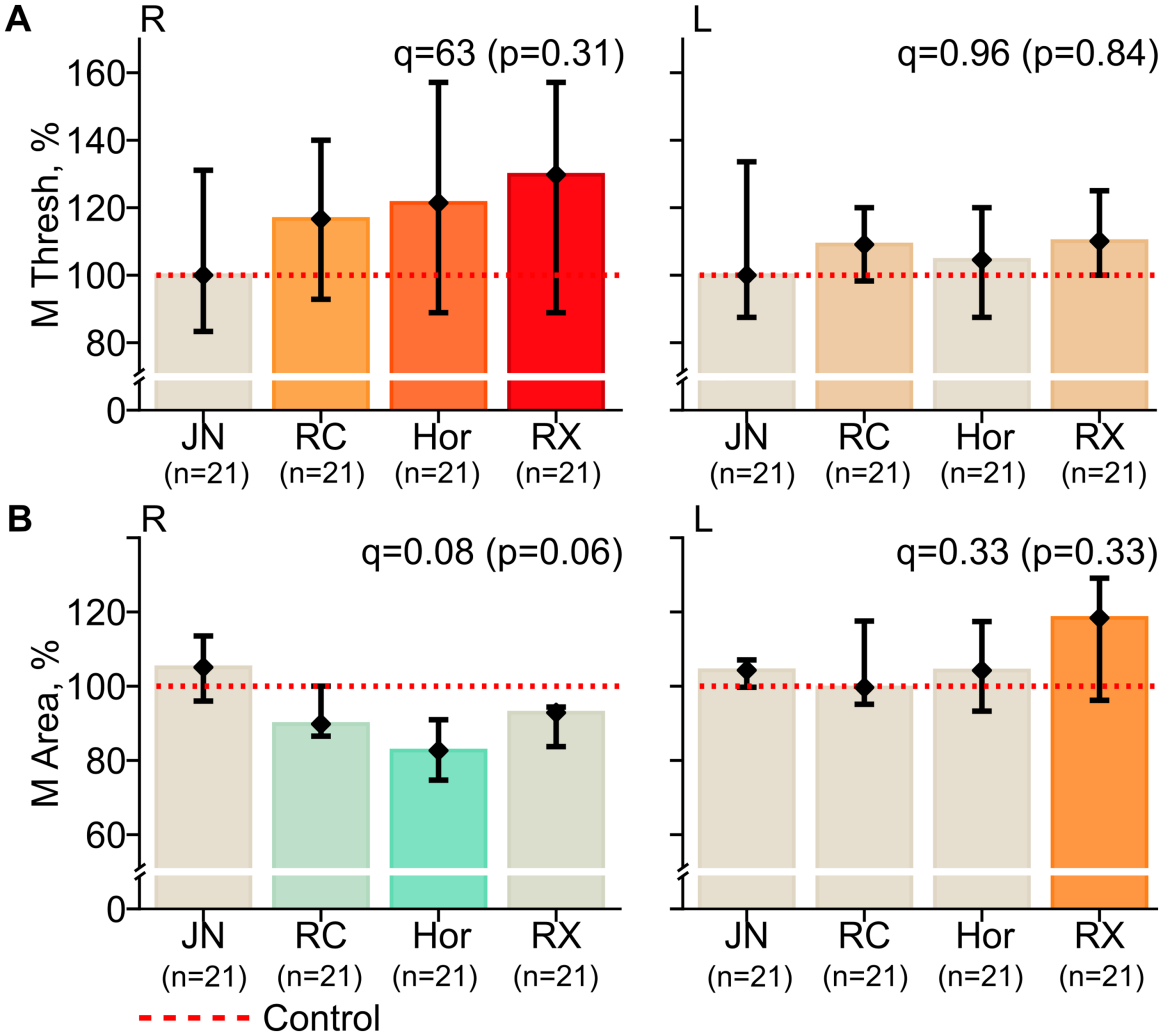
Threshold and area of the soleus M-response under different conditions for the right (R) and left (L) legs **(A)** M-response threshold values of the soleus muscle **(B)** Area of the soleus M-response. JN = Jendrassik maneuver (*n* = 21 participants); RC = virtual reality (VR) exposure—“Roller coaster” scenario (*n* = 21); Hor = VR “Horror” scenario (*n* = 21); RX = VR “Relaxation” scenario (*n* = 21). All comparisons included *n* 21 participants.

Figure 3A shows the distribution of M-response thresholds of the *m. soleus* under different experimental conditions. In the right (dominant) leg, no significant effects were found when comparing M-response thresholds (*q* = 0.63, *p* = 0.31). Although values fluctuated near the control level across all conditions, there was a tendency toward increased thresholds in all VR-presentation groups. In the left (non-dominant) leg, no significant differences were observed between conditions either (*q* = 0.96, *p* = 0.84).

When assessing the *soleus* M-response area (Figure 3B) in the right leg, a trend toward a difference was observed (*q* = 0.08, *p* = 0.06). This trend was driven by lower M-area values under the roller-coaster video 89.81% (90% CI: 85.9; 98.0), and the horror video 82.6% (90% CI: 74.7; 90.9). In the left leg, no significant differences were found between conditions (*q* = 0.33, *p* = 0.33). Thus, despite some visual fluctuations, the mean M-response area values remained close to the control range in most cases.

### Changes in RR-interval and RMSSD

Table 1 presents data on R-R interval duration and RMSSD at rest and during exposure to various VR videos. Statistical analysis revealed small differences between the control condition and the experimental stimuli.

**Table 1:** Changes in RR-interval and RMSSD.

Condition	RR interval, Md (90% CI), s	*p*‑value
RR interval during at rest (Control) and VR, Md (90% CI), s		
Control	0.79 (0.73; 0.84)	Overall: *p* ≤ 0.05^f^ Control vs. RC: *p* ≤ 0.05^w^ Control vs. Hor: *p* ≤ 0.05^w^ Control vs. RX: *p* = 0.45^w^
RC	0.70 (0.64; 0.71)	
Hor	0.73 (0.72; 0.79)	
RX	0.75 (0.71; 0.81)	
Condition	RMSSD, Md (90% CI)	*p*‑value
RMSSD during at rest (Control) and VR		
Control	61.0 (50.5; 83.5)	*p* = 0.15^f^
RC	70.6 (53.9; 77.6)	
Hor	58.2 (44.9; 82.15)	
RX	47.9 (38.9; 59.5)	

RC, roller-coaster; Hor, horror; RX, relax; ^f^The Friedman test (chi-square based) and ^w^Wilcoxon signed-rank test.

Analysis of autonomic responses showed a significant effect of the virtual stimulus on RR interval duration (*p* ≤ 0.05). The most pronounced decrease in the RR interval occurred during the dynamic “roller coaster” video: 0.70 s (90% CI: 0.64; 0.71) vs. the control value of 0.79 s (90% CI: 0.73; 0.84), indicating an increase in heart rate under dynamic VR stimulation. In contrast, the vagal modulation index (RMSSD) did not show significant between-condition differences (*p* = 0.15).

## Discussion

This preliminary research provides evidence that specific immersive audiovisual content delivered via a virtual reality headset can selectively modulate segmental reflex excitability. This effect was primarily seen in H-reflex amplitude, whereas H-reflex thresholds remained unchanged. This pattern suggests that the observed changes do not arise from altered afferent input excitability *per se*. Instead, they likely reflect modulation of motoneuron recruitment and synaptic efficacy within the Ia-afferent pathway. In particular, the absence of significant H-reflex threshold changes in either limb suggests that baseline Ia-afferent excitability is stable. This is consistent with the view that threshold parameters are less sensitive to brief sensory and emotional stimuli than amplitude-based measures (Knikou, 2008; Kuck et al., 2018).

The most pronounced changes were associated with emotionally charged VR stimuli. Viewing the “horror” video reduced H-reflex amplitude. A decrease in H-reflex amplitude is traditionally interpreted as increased presynaptic inhibition of Ia afferents or enhanced descending inhibitory drive (Xu et al., 2022; Metz et al., 2023). This suggests that an emotionally salient sensory context can modulate segmental reflex excitability without altering baseline reflex thresholds. Similar effects have been reported in threat simulations within virtual environments, where H-reflex amplitude decreased without changes in background muscle activity (Grosprêtre et al., 2023). These observations suggest the central origin of the modulation and that the changes likely arise from a redistribution of descending control under sensory and emotional influence.

In contrast, the H-reflex reduction observed in roller coaster conditions may be due to other mechanisms: strong visual motion and vestibular conflict may cause this effect through sensory integration and autonomic mechanisms, and not just through fear-related emotion processing.

These findings are also consistent with the concept that emotionally salient sensory information can influence motor output through interactions between limbic, cortical, and descending motor pathways (Allen et al., 2021; Bringman et al., 2022).

In contrast to VR stimuli, the Jendrassik maneuver increased H-reflex amplitude in both limbs. This finding aligns with classical neurophysiological principles regarding the reduction of presynaptic inhibition and enhanced motoneuron recruitment during voluntary co-contraction (Misiaszek, 2003; Oza et al., 2017; Grosprêtre et al., 2018). It also validates our experimental paradigm and serves as an internal physiological control.

Analysis of M-response parameters revealed no significant changes under any condition, suggesting that direct motor excitability remains relatively stable and that the observed H-reflex changes are not due to altered efferent fiber conductivity or general motoneuron excitability. Hence, the effects of VR stimulation can be interpreted as predominantly reflex-mediated.

The trend toward a reduced M-response area in the right leg during emotional video conditions did not reach statistical significance. This may reflect variability in motor responses or the influence of non-specific factors such as attention and sensory load. However, the absence of reliable changes precludes further interpretation of efferent involvement.

Notably, the relaxation condition did not significantly modulate H-reflex amplitude. This null result may reflect a physiological floor effect (participants were already relaxed at baseline), limited sensitivity of relatively more passive, meditative videos to induce measurable spinal changes, or restricted statistical power. Nevertheless, the absence of modulation under relaxing conditions can provide a meaningful contrast to the suppressive effects observed during stimuli designed to elicit more charged emotional responses.

### Limitations

Importantly, this study did not directly assess subjective emotional state (e.g., valence, arousal, fear, relaxation) or more sensitive autonomic measures (skin conductance). This limits our interpretation of the VR stimuli as strictly affective. Furthermore, the video stimuli differed not only in emotional valence but also in sensory complexity and dynamics. Consequently, the present design does not allow us to fully disentangle the effects of emotional content from those of multisensory stimulation. It should also be acknowledged that background EMG was not monitored. Given the visually dynamic nature of the VR stimulation, some degree of uncontrolled muscle activation may have subtly influenced the assessment of motor pool excitability and remained unaccounted for. Additionally, it should be noted that this is a preliminary study designed to inform future investigations; it was not substantially powered for confirmatory inference, and all observed effects should be considered as preliminary results. Finally, while we implemented a Latin square randomization to minimize order effects and verified baseline stability before each condition, we did not conduct a formal statistical analysis of potential carry-over or sequence effects. Future studies with larger samples should explicitly model order as a factor to confirm the robustness of the observed VR-induced modulation.

## Conclusion

Immersive audiovisual stimuli with content designed to elicit emotional responses, as tested in this study, can modulate spinal reflex activity, as reflected by changes in H-reflex amplitude. This effect indicates selective engagement of reflex-regulating mechanisms at the level of Ia-afferent transmission and descending influences, without altering general motor or autonomic excitability. The results of this preliminary research provide a foundation for the design of future investigations.

## Data Availability

The raw data supporting the conclusions of this article will be made available by the authors, without undue reservation.
